# Effects of Transcranial Direct Current Stimulation on the Control of Finger Force during Dexterous Manipulation in Healthy Older Adults

**DOI:** 10.1371/journal.pone.0124137

**Published:** 2015-04-09

**Authors:** Pranav J. Parikh, Kelly J. Cole

**Affiliations:** 1 School of Biological and Health Systems Engineering, Arizona State University, Tempe, AZ, 85226, United States of America; 2 Motor Control Laboratories, Department of Health and Human Physiology, University of Iowa, IA 52242, United States of America; University of California, Merced, UNITED STATES

## Abstract

The contribution of poor finger force control to age-related decline in manual dexterity is above and beyond ubiquitous behavioral slowing. Altered control of the finger forces can impart unwanted torque on the object affecting its orientation, thus impairing manual performance. Anodal transcranial direct current stimulation (tDCS) over primary motor cortex (M1) has been shown to improve the performance speed on manual tasks in older adults. However, the effects of anodal tDCS over M1 on the finger force control during object manipulation in older adults remain to be fully explored. Here we determined the effects of anodal tDCS over M1 on the control of grip force in older adults while they manipulated an object with an uncertain mechanical property. Eight healthy older adults were instructed to grip and lift an object whose contact surfaces were unexpectedly made more or less slippery across trials using acetate and sandpaper surfaces, respectively. Subjects performed this task before and after receiving anodal or sham tDCS over M1 on two separate sessions using a cross-over design. We found that older adults used significantly lower grip force following anodal tDCS compared to sham tDCS. Friction measured at the finger-object interface remained invariant after anodal and sham tDCS. These findings suggest that anodal tDCS over M1 improved the control of grip force during object manipulation in healthy older adults. Although the cortical networks for representing objects and manipulative actions are complex, the reduction in grip force following anodal tDCS over M1 might be due to a cortical excitation yielding improved processing of object-specific sensory information and its integration with the motor commands for production of manipulative forces. Our findings indicate that tDCS has a potential to improve the control of finger force during dexterous manipulation in older adults.

## Introduction

Aging often impairs the ability to perform dexterous manipulation. Poor finger force control seems likely to contribute to this impairment, and may partially explain ubiquitous age-related behavioral slowing [1,2]. Adults over 60 years demonstrate altered control of the magnitude and direction of finger forces applied to the object during functionally-relevant tasks [2–5]. Moreover, older adults usually apply greater grip force on the object than younger adults during object manipulation [3,6]. Exertion of unnecessarily greater grip force can be detrimental to task performance because small finger misalignments can lead to unwanted torque on the object, thus affecting its orientation [3]. Improving older adults’ ability to control grip forces may improve manual performance on skillful tasks.

Our work has shown that repeated manipulation of an object can reduce the grip force, thus assisting older adults in reducing the amount of unwanted torque applied to the object [3]. However, the effect of motor practice on the grip force control was mainly observed when the mechanical properties of the object (size, weight, surface friction) remained constant. In contrast, when the object’s mechanical properties were uncertain, it has been reported that older adults continue to exert greater grip force on the object even after repeated manipulation [1,6–8]. This finding might indicate a deficit in processing sensory signals from tactile afferents of cutaneous receptors in the fingertips [1,3,6].

Recently, delivery of transcranial direct (low-intensity) anodal current (anodal tDCS) over motor cortex (M1) in synchrony with practice or training has been shown to facilitate acquisition of a new skill, as well as retention of an acquired skill or performance [9–11]. However, the effects of anodal tDCS over M1 on the altered control of finger forces during object manipulation by older adults have not been fully determined [9,12]. The purpose of this study was to determine the effects of enhancing the activity of M1 using anodal tDCS on the control of grip forces in healthy older adults while they manipulated an object with unpredictable mechanical properties. Specifically, we instructed older adults to grip and lift an object whose slipperiness at the grasp surfaces was unpredictably changed between trials. Making the object’s surface more or less slippery in an unpredictable fashion would necessitate online processing of sensory information about friction at the hand-object interface for accurate scaling of grip force on a trial-by-trial basis [13,14]. It is known that M1 plays a role in motor adaptation/learning that requires integration of feedback information for modulating motor commands for the control of finger forces [15–17]. We hypothesized that anodal tDCS over contralateral M1 would facilitate the use of feedback information about surface friction for generating the motor commands for grip force application, *as indicated by a reduction in the magnitude of grip force exerted on the object*. The data presented here was obtained during experimental sessions described in previously reported work [9]. However, the data reported in the current study has not been published elsewhere.

## Materials and Methods

### Subjects

Eight right-handed (self-reported) healthy older adults (75 ± 8 years [mean ± SD]; 5 males) were recruited. The following exclusion criteria were utilized to select a group of healthy older adults: (a) injury or disease of the brain, (b) injury or disease affecting the hands or arms, (c) wrist or hand pain that requires daily prescription medication, (d) diabetes, (e) high blood pressure requiring medication, (f) sensory disturbances of the dominant arm, (g) corrected vision worse than 20/20, (h) history of heart disease, (i) implanted battery-driven devices such as implanted pacemakers, defibrillators, infusion pumps, (j) family history of epilepsy, and (k) presence of metal in skull. All participants appeared to be aware of their surroundings and current events based on their responses to questions designed to screen for impaired cognitive status [1,3,9,18]. We tested participant’s ability to sense vibration applied to the distal interphalangeal joint of the right index finger was tested using the Rydel-Seiffer graduated tuning fork (Arno Barthelmes, Tuttlingen, Germany) [9] to rule out undiagnosed sensory neuropathy. Participants with a vibration threshold within the limits of published norms for their age [19] were invited to provide informed consent and participate in the study. Subjects completed a Transcranial Magnetic Stimulation (TMS) adult safety screen questionnaire to confirm their eligibility to participate in the TMS procedure [20]. The tactile thresholds findings were reported in our previous work [9]. The University of Iowa Human Subject Internal Review Board approved the experiment and written informed consent was obtained from all subjects according to the Declaration of Helsinki.

### Apparatus

#### Grip-lift object

**Fig 1:**
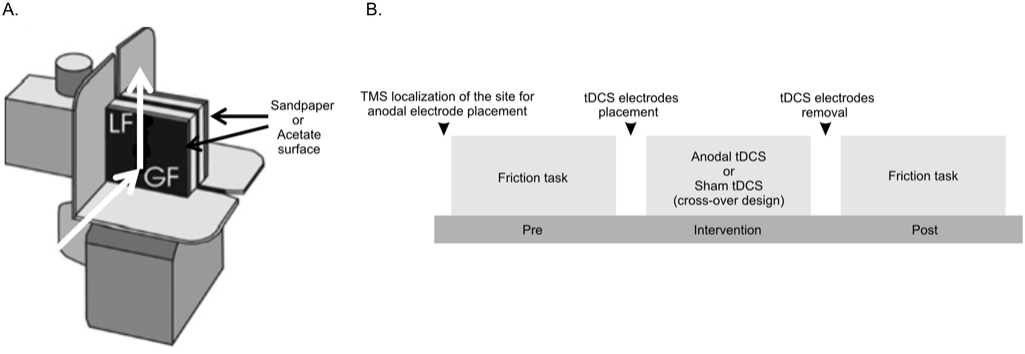
The test object and the experimental procedure. (A) Grip-lift object: Strain gauges embedded in the object measured grip force and load force at contact surfaces of both digits. The contact surfaces were covered either with acetate or sandpaper surface. (B) Experimental procedure: The intervention was delivered for 20 minutes. Subjects practiced a motor task during the intervention period.

The test object (Fig 1A; 4N) had two opposing contact surfaces (35 by 35 mm) parallel to each other, with a separation of 2.2 cm between the digit contact surfaces [6,18,21]. The surfaces were covered with either black sandpaper (grit-320) or black acetate. Strain gauges integrated in the object measured the force acting perpendicular to the contact surfaces (grip force) and the vertical tangential (load) force separately at both contact surfaces. An accelerometer (SenSyn SXL010G) affixed to the object measured the vertical acceleration.

#### Experimental task

The task adopted in the current study was similar to the task used in [6]. Subjects were instructed to grasp the object using thumb and index fingers, to lift the novel object ~4 inches above the table with elbow flexion, hold it for 4 sec, and then replace it on the table. Subjects practiced the grip and lift task a few times. For practice trials, the contact surfaces were covered with sandpaper. After initial familiarization, the slipperiness of the object was pseudo-randomly varied across 25 trials by using either acetate or sandpaper coverings at the two gripping surfaces. On 6 additional trials, subjects were instructed to lift the object to a stationary position, and then slowly to relax their grip to allow the object to slip between their fingers. These slips were used to estimate the inverse coefficient of static friction at each digit (see *Data Analysis*). In total, subjects performed these trials with ~5 seconds of rest period in between trials.

#### Experimental Procedure

Each subject participated in two experimental sessions [9]. During each session, subjects were instructed to perform the grip and lift task described above (Fig 1B). The grip and lift task was performed in dim light to prevent visual discrimination of the surface. Subjects washed their hands with soap and water. They sat in front of the table with their dominant hand resting on their thigh. Following the baseline (pre) performance of the grip and lift task, subjects received either anodal tDCS or sham tDCS to the primary motor cortex contralateral to the dominant hand, in combination with motor practice. Each subject received anodal tDCS and sham tDCS in two sessions separated by more than 5 days, each lasting 2 hours. During anodal tDCS and sham tDCS, subjects were instructed to practice the Grooved Pegboard test six times during the stimulation period. This study was part of a larger study where subjects performed several manual tasks prior to and following anodal or sham tDCS (refer [9]). We delivered anodal or sham tDCS during the Grooved Pegboard test because anodal tDCS has been shown to improve manual performance when administered during tasks requiring dexterous manipulation that are part of the Jebsen-Taylor test [11]. Fifty percent of the subjects first received anodal tDCS, while the other 50% received sham tDCS during their first session. Subjects were not aware of the order of this presentation and thus this is a single-blinded study [9]. After the stimulation, subjects re-performed the grip and lift task. In addition to the grip and lift task, subjects also performed other object manipulation tasks before and after tDCS as reported in [9] during the 2-hour session. The order of presentation of these tasks was identical across anodal and sham tDCS sessions, thus the extent to which performance of one task may influence that of another task would be similar across anodal and sham tDCS sessions. Participants were explicitly asked at the end of second experimental session whether they could ascertain which session involved stimulation and which session involved sham stimulation.

#### Transcranial Direct Current Stimulation (tDCS)

tDCS was applied via 2 conducting 25 cm^2^ saline-soaked sponge electrodes. Anodal and cathodal electrodes were placed on the scalp overlying the left primary motor cortex hand area and the right supraorbital region, respectively [9]. A constant current at an intensity of 1mA (current density: 0.04 mA/cm^2^; total charge: 0.048 C/cm^2^) was applied for 20 min in the anodal tDCS group and for up to 30 sec in the Sham session [9]. A single-pulse Transcranial Magnetic Stimulation (TMS) technique was used to estimate the site of tDCS anodal electrode placement at the beginning of each session. TMS was applied using a figure-of-eight coil with a 7 cm diameter (Magstim Company Ltd., Whitland, Dyfed, UK). Using suprathreshold TMS pulses, we located the region of the left motor cortex that represented the right first dorsal interosseus muscle (FDI). The location of tDCS anodal electrode placement was determined as a site where a reliable visible muscle twitch was evoked using TMS (Fig 1B).

### Data Analysis

Force and acceleration signals were acquired at 500 samples per second with a 16-bit resolution, and analyzed with a personal computer running Datapac software (Datapac 2000 v 2.0 RUN Technologies, Mission Viejo, California).

The magnitude of grip force was defined as an average of normal (Fn) forces applied to the object at each contact surface (i.e. {Fn_thumb_ + Fn_finger_} ÷ 2). The load force was calculated by summing the vertical tangential force at both digits (tangential thumb + tangential finger). Trials in which participants performed slips provided data for estimating the inverse coefficient of static friction (normal force_slipping digit_/load force_slipping digit_) [6]. These trials were not included in other analysis of fingertip forces. Grip force at the time of object lift-off, the primary dependent variable, was measured for all trials. The moment of object lift-off from the table surface was determined as the time when the acceleration signal increased to twice the standard deviation above baseline noise, and remained above that criterion level for at least 50ms. We also measured the load phase duration that initiated with a sustained increase in lift force rate and ended with the lift-off of the object from the support surface [6]. We found that all data were normally distributed (Shapiro-Wilk test: all p-values>0.2). The grip force at the lift-off, the load phase duration, and the inverse coefficient of friction were tested using repeated measures ANOVA with Intervention (Anodal tDCS, Sham tDCS), Time (Pre, Post), and Texture (Acetate, Sandapaper) as within-subject factors. Post-hoc comparisons were performed using two-tailed paired t-test with appropriate Bonferroni correction. All statistical analyses were performed using SPSS (version 19.0, IBM). Significance level for all statistical analyses was set at α = 0.05. All values in the text, figures, and the table represent group mean ± 1 standard error of measurement unless stated otherwise.

## Results

All participants completed the study with no adverse effects. Each subject self-reported mild tingling or burning skin sensations under the stimulating electrodes for the initial ~30 sec of each anodal and sham stimulation period, followed by no sensation for the remainder of the stimulation period, as has been reported [22]. As reported in Parikh and Cole [9], none of the participants were able to distinguish between anodal and sham tDCS.

**Fig 2:**
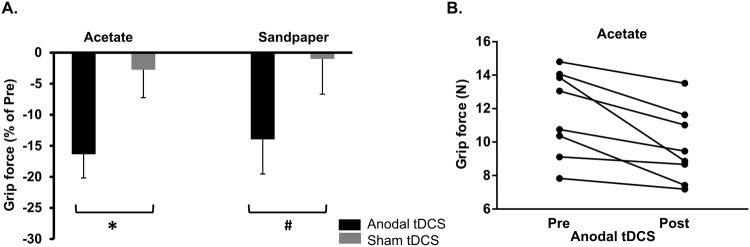
Effects of tDCS on the grip force measured at lift-onset. (A) Changes in the grip force (in %) following anodal and sham tDCS measured with respect to the baseline (pre) measurements for both acetate and sandpaper surfaces. Asterisk indicates p<0.025. # indicates p<0.1. (B) Subject-wise grip force (N) data averaged across acetate trials obtained before (pre) and after (post) anodal tDCS.

We found that the subjects significantly reduced the magnitude of grip force exerted on the object at lift-onset following anodal compared to sham tDCS (Significant Intervention × Time interaction: F_1,7_ = 7.011; p = 0.033; η^2^ = 0.5; Fig 2). Subjects practice a motor task (i.e. the Grooved Pegboard test) during both anodal and sham tDCS. Thus, the above finding suggests that the contribution of motor practice in significantly reducing the grip force following anodal tDCS can be ruled out. For the acetate condition, the reduction in grip force from pre- to post-intervention was significantly greater following anodal than sham tDCS (-16.3% versus 2.7%; posthoc paired t-test: t_7_ = -2.9; p = 0.02; Fig 2A). This reduction in grip force for acetate trials following anodal tDCS was significantly different than zero, and was exhibited by *all* subjects (Fig 2B; one-sample t-test: p = 0.004). For sandpaper trials, we found that the reduction in grip force measured at lift-onset from pre- to post-intervention was marginally different between anodal and sham tDCS (-13.9% versus -1.02%; posthoc paired t-test: t_7_ = -1.95; p = 0.09; Fig 2A). Moreover, the effect of Intervention (anodal versus sham tDCS) on the magnitude of grip force was not different across the two texture trials (No Intervention x Texture interaction: F_1,7_ = 1.6; p = 0.25). We found no difference in baseline grip force (i.e. pre; Fig 1) between anodal and sham tDCS for both acetate and sandpaper trials (both p-values>0.2). Subjects exerted significantly greater grip force on the object at lift-onset for acetate compared to sandpaper trials (main effect of Texture: F_1,7_ = 9.398; p = 0.018; η^2^ = 0.57; Table 1). We did not observe a Time x Texture interaction (F_1,7_ = 1.2; p = 0.31) nor Intervention x Time x Texture interaction (F_1,7_ = 0.52; p = 0.49).

**Table 1:** Grip force (N) and loading phase duration (ms) for both acetate and sandpaper surfaces measured before (pre) and after (post) anodal and sham tDCS.

		Acetate		Sandpaper	
		Anodal	Sham	Anodal	Sham
**Grip Force (N)**	Pre	11.7 ± 0.9	10.5 ± 0.94	10.2 ± 0.8	9.36 ± 0.95
	Post	9.7 ± 0.8	10.1 ± 0.9	8.6 ± 0.65	9.06 ± 0.74
**Loading phase duration (ms)**	Pre	201.03 ± 33.61	199.13 ± 28.2	178.36 ± 24.9	198.275 ± 32.7
	Post	193 ± 34.9	177.3 ± 29.25	202 ± 47.5	168.5 ± 26

**Fig 3:**
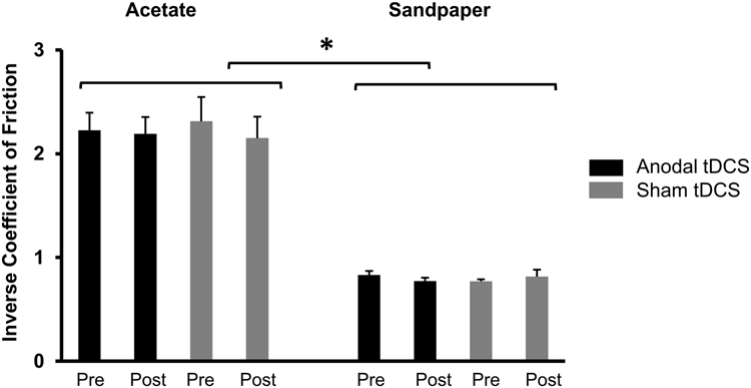
Inverse coefficient of friction for acetate and sandpaper surfaces measured before (pre) and after (post) anodal and sham tDCS. Asterisk indicates p<0.001.

As expected, the inverse coefficient of friction was significantly greater for acetate than sandpaper trials (Significant mean effect of Texture: F_1,7_ = 84.1; p<0.001; η^2^ = 0.92). There was no change in the inverse coefficient of friction following anodal and sham tDCS across two conditions (No Significant Intervention × Time interaction: p = 0.93; Fig 3). We found no difference in baseline coefficient of friction between anodal and sham tDCS for both acetate and sandpaper trials (both p-values>0.15).

The loading phase duration for acetate trials were not different than those for sandpaper trials (No main effect of Texture: F_1,7_ = 1.96; p = 0.2; Table 1). We found no change in the loading phase duration across two conditions following anodal and sham tDCS (No Significant Intervention × Time interaction: p = 0.13). Prior to intervention, loading phase duration was not different between anodal and sham tDCS for both texture conditions (both p-values>0.1).

## Discussion

We determined the effects of anodal tDCS on the control of finger forces during object grasping and manipulation in a healthy group of older individuals. The novel finding from this study is that a single session of anodal transcranial direct current stimulation in healthy older adults significantly improved the control of grip force during object manipulation in an unpredictable environment. Specifically, anodal tDCS over M1 reduced the magnitude of grip force applied to the object with uncertain slipperiness, when compared with sham tDCS.

Healthy aging impairs fine control of finger forces during object grasping and manipulation. Impairment in finger force control in older adults include biased fingertip force vectors during simple finger pressing task [23], overexertion of grip force on the object [1,3,6,8,23], delayed or altered force response to unexpected perturbations due to impoverished sensory function [6,7,23,24], increased force in non-instructed direction during isometric force production task [5], impaired dynamic control of finger forces during a strength-dexterity paradigm [4,25] reduced ability to produce smooth forces during isometric force production tasks [26,27] and in a key-slot task [3], and impaired control of rotational force applied to the object during functionally-relevant grip and lift task [3]. Poor control of magnitude and direction of finger forces can alter the orientation of hand-held object resulting in impairment in dexterous manipulation. We found that a single session of anodal tDCS over M1 significantly reduced the magnitude of grip force exerted on the object whose slipperiness at graspable surfaces was unexpectedly changed across trials. Because the acetate surface resulted in lower friction than the sandpaper surface at the skin-object interface, older adults used significantly greater grip force to manipulate the object covered with acetate than sandpaper prior to anodal and sham tDCS. This provided a greater margin for reduction in the grip force following anodal tDCS for the acetate trials. For the sandpaper trials, failure to observe unequivocal reduction in the grip force following anodal tDCS might be due to this ‘floor’ effect. Importantly, the reduction in the grip force exerted on the object can potentially assist older adults in reducing unwanted torque exerted on the object from the misalignment of digits and their force vectors [3]. Minimizing unwanted torque on the object can assist in maintaining its orientation during manipulation in older adults, and the reduced need to correct orientation errors may speed motor performance as well [2,3]. Our findings showed that low intensity electrical stimulation of M1 for 20 minutes can help older adults in improving the control of finger forces, potentially improving dexterous manipulation.

Anodal tDCS-induced reduction in the grip force in the current study might have resulted from the facilitation of sensorimotor integration during object manipulation. Dexterous manipulation of an object with changing slipperiness in an unexpected fashion required online processing of sensory information regarding surface friction for accurate modulation of motor command for force generation on a trial-to-trial basis. Furthermore, it is well-known that anodal tDCS increases excitability of cortical neurons underneath the anodal electrode (i.e. M1 neurons in the current study) through long-term potentiation-like mechanism that depends on NMDA and TrkB receptor activation [28,29]. Thus, the increase in neuronal excitability following anodal tDCS over M1 might have improved the integration of feedback information about surface friction on a trial-to-trial basis for accurate force application. This interpretation is consistent with the role of M1 in motor adaptation and performance during grasping and manipulation [9,15,30]. Furthermore, anodal tDCS is known to influence distant connections of the area being stimulated [31,32]. In our study, stimulation of M1 using tDCS might have stimulated the primary somatosensory area (S1) involved in processing the incoming tactile feedback regarding surface friction through horizontal cortico-cortical connections [33,34]. In addition, the large size (25 mm^2^) of the anodal tDCS electrodes might have directly increased the excitability of S1. Thus, simultaneous stimulation of M1 and S1 might have played a role in improving sensorimotor integration allowing older adults to reduce their grip force during manipulation of an object with an uncertain property, i.e. slipperiness.

The reduction in grip force magnitude did not seem to have resulted from the change in tactile sensitivity to the surface texture. As reported in Parikh and Cole [9], tactile thresholds measured using Semmes-Weinstein pressure filaments did not change following anodal stimulation over M1. Moreover, overexertion of grip force during object manipulation observed in older adults might not be associated with the age-related tactile deterioration [3]. We have previously reported that repeated practice of a grip and lift task using an object with a constant mechanical property in older adults reduced the magnitude of grip force exerted on the object [3]. This finding ruled out the primary role of age-related tactile impairment in overexertion of grip force [3,35] because subjects with digital anesthesia persistently used greater grip force during multiple grip and lift tasks [13,36,37].

We want to acknowledge a few caveats of the current study. The small sample size of healthy older adults, who lacked the typical comorbidities of old age limits the generalizability of the observed findings to the general population of old adults. However, *all older participants* responded similarly to anodal tDCS, which conveys robustness to our finding (Fig 2B). Nevertheless, it may be that certain comorbidities may affect the capacity of the brain to respond to the stimulation, and further studies to confirm generalizability of the observed effects of anodal tDCS on finger force control in larger sample of older adults are needed. Also our study did not include a younger control group. Thus, we are unable to comment on whether the grip force exerted following anodal stimulation by older adults approached the low force levels observed in the younger population. It is likely that anodal tDCS facilitates sensorimotor integration in young adults as well. However, we may not observe significant reduction in grip force following anodal tDCS in young adults. Young adults have been previously found to produce significantly lower grip force when compared with older adults during manipulation of an object with unexpected slipperiness [6]. Therefore, the ability of improved sensorimotor integration due to anodal tDCS to further lower grip force in young adults will approach the physical limits in terms of the minimal grip force required to avoid slips [6,38]. Overall, although the finding of reduction in grip force might be specific to older adults, the lack of younger controls does not undermine our interpretation that anodal tDCS enhanced sensorimotor integration in older adults.

Overall, using a crossover and sham-controlled study, we have shown that anodal tDCS in older adults can reduce the grip force exerted on an object with an uncertain mechanical property during grasping and dexterous manipulation, potentially due to improved sensorimotor integration. Thus, we demonstrated that tDCS has a potential to ameliorate the age-related impairment in the control of finger force during grasping and manipulation. Future studies will assess the persistence of the observed effects over several hours or days.
